# Uptake, transport, distribution and Bio-effects of SiO_2_ nanoparticles in Bt-transgenic cotton

**DOI:** 10.1186/s12951-014-0050-8

**Published:** 2014-12-05

**Authors:** Van Nhan Le, Yukui Rui, Xin Gui, Xuguang Li, Shutong Liu, Yaning Han

**Affiliations:** College of Resources and Environmental Sciences, China Agricultural University, Yuanmingyuan Xilu No.2, Haidian District Beijing, 100193 China; Research Institute for Aquaculture No.1, Tu Son, Bac Ninh 222260 Viet Nam

**Keywords:** SiO_2_ nanoparticles, Bt-transgenic cotton, Transport, Distribution, Xylem sap, Phytotoxicity

## Abstract

**Background:**

SiO_2_ nanoparticle is one of the most popular nanomaterial which has been used in various fields, such as wastewater treatment, environmental remediation, food processing, industrial and household applications, biomedicine, disease labeling, and biosensor, etc. In agriculture, the use of SiO_2_ nanoparticles as insecticide, carriers in drug delivery, or in uptake and translocation of nutrient elements, etc., has been given attention. However, the effects of nanoparticles on plants have been seldom studied. In this work, the toxicity of SiO_2_ nanoparticles and their uptake, transport, distribution and bio-effects have been investigated in Bt-transgenic cotton.

**Methods:**

The phytotoxic effects of SiO_2_ nanoparticles were exhibited in Bt-transgenic cotton with different SiO_2_ concentrations of 0, 10, 100, 500 and 2000 mg.L^−1^ for 3 weeks through dry biomasses, nutrient elements, xylem sap, enzymes activities, and hormone concentrations. The uptake and distribution of nanoparticles by the plants were confirmed using transmission electron microscopy (TEM).

**Results:**

The SiO_2_ nanoparticles decreased significantly the plant height, shoot and root biomasses; the SiO_2_ nanoparticles also affected the contents of Cu, Mg in shoots and Na in roots of transgenic cotton; and SOD activity and IAA concentration were significantly influenced by SiO_2_ nanoparticles. In addition, SiO_2_ nanoparticles were present in the xylem sap and roots as examined by TEM showing that the SiO_2_ nanoparticles were transported from roots to shoots via xylem sap.

**Conclusions:**

This is the first report of the transportation of SiO_2_ nanoparticles via xylem sap within Bt-transgenic cotton. This study provides direct evidence for the bioaccumulation of SiO_2_ nanoparticles in plants, which shows the potential risks of SiO_2_ nanoparticles impact on food crops and human health.

## Background

Nanoparticles (NPs), which have at least one dimension in 1–100 nm range [1,2], can dramatically modify the physico-chemical properties when compared to bulk materials [3]. In recent years, nanotechnology has been significantly developed and expanded to various fields, such as water purification, wastewater treatment, environmental remediation, food processing, packaging, industrial and household purposes, biomedicine, ceramics synthesis, disease labeling, drug deliver, and biosensor, etc. [4-6]. SiO_2_ nanoparticle is one of the most popular nanomaterial which has been used in these fields. On the other hand, Siddiqui and Al-Whaibi [7] reported that application of nano-SiO_2_ enhanced significantly the characteristic of seed germination of tomato (*Lycopersicum esculentum Mill. Cv Super strain B*). In agriculture, through pharmaceutical applications that mesoporous SiO_2_ NPs are used as controlled release carriers in drug delivery Chen *et al.* [8]. According to Marmiroli *et al.* [9] Silicon was applied on uptake and translocation of Arsenic in tomato (*Solanum lycopersicum L.*). Debnath *et al.* [10] proposed the application of surface functionalized nano-SiO_2_ as an insecticide to protect agricultural products by overcoming the resistance to conventional insecticides. More and more attention has been paid on the ecological safety on microorganisms [11], animals [12] and plants [13] regarding the application of NPs.

According to Monica and Crenomini [14], the effect of nanomaterials on plants can be positive or negative. Zheng *et al.* [15] indicated that spinach seeds treated by nano-TiO_2_ had 73% more dry weight, three times higher in photosynthetic rate compared to the control over germination period of 30 days. Khodakovskaya *et al.* [16] reported that MWCNTs increased the seed germination of tomato up to 90% when compared to 71% in control treatment. With the rapid development of nanotechnology, there is a growing concern among scientists and regulatory agencies about its potential negative impacts on human health and environment. The phytotoxicity is one of the concerns for nanomaterial applications, and the level of phytotoxicity depends on the types of nanomaterials and its potential applications [6]. The toxicity of nanomaterials was emerging studied and basically evidence several negative effects on growth and development of plantlets [14]. According to USEPA [17], studies on seed germination and root elongation are often accompanied by other biomass changes and anatomical-histological evaluations. This could be considered as evidence of in situ toxicity symptoms. Yang and Watts [18] reported that nanoparticles cause negative effects on root elongation in the plant species corn, cucumber, soybean, cabbage and carrot. Lin and Xing [19] observed the phytotoxicity of nanoparticles in multiwall carbon nanotube; aluminum, alumina, zinc and zinc oxide on seed germination and root growth of radish, rape canola, ryegrass, lettuce, corn and cucumber. The germination of ryegrass and corn were inhibited by 2000 mg.L^−1^ nano-size Zn (35 nm) and ZnO (20 nm) treatments, respectively. The root elongation of studied plants were also affected when suspended in 2000 mg.L^−1^ nano-size Zn and ZnO.

The first transgenic plants were researched in the early of 1980s, and then the transgenic technology has extremely quickly developed. In 1996, the transgenic crops were 1.7 million hectares and planted in six countries in the world. And this figure reached 90 million hectares in 2005 [20]. The main transgenic plants were corn, soybean, canola, and cotton; therein cotton is one of the major fibre crops has global significance, which cultivated in tropical and subtropical regions in more than eighty countries [21]. It took China of more than 10 years of researches in demonstration, extension and final commercialization of Bt transgenic cotton. The inbuilt genetic resistance to bollworms is very effective in controlling the yield loss caused by bollworms ([22-24], [25]). Today, the Bt transgenic cotton is widely used by farmers and this makes China the largest cotton producing country in the world [22]. However, the ecological risk become a big concern in transgenic plants as more and more new nanomaterials have been developed and applied in transgenic plants with unpredictable results, which need to be studied.

In this study, we investigated the toxicity of SiO_2_ NPs to non-transgenic and Bt-transgenic cotton and their uptake, transport, distribution and bio-effects in Bt-transgenic cotton through dry biomasses, nutrient elements, xylem sap, enzymes activities, hormone concentrations and distribution of SiO_2_ in the roots.

## Results and discussion

### SiO_2_ nanoparticle characterization

**Figure 1:**
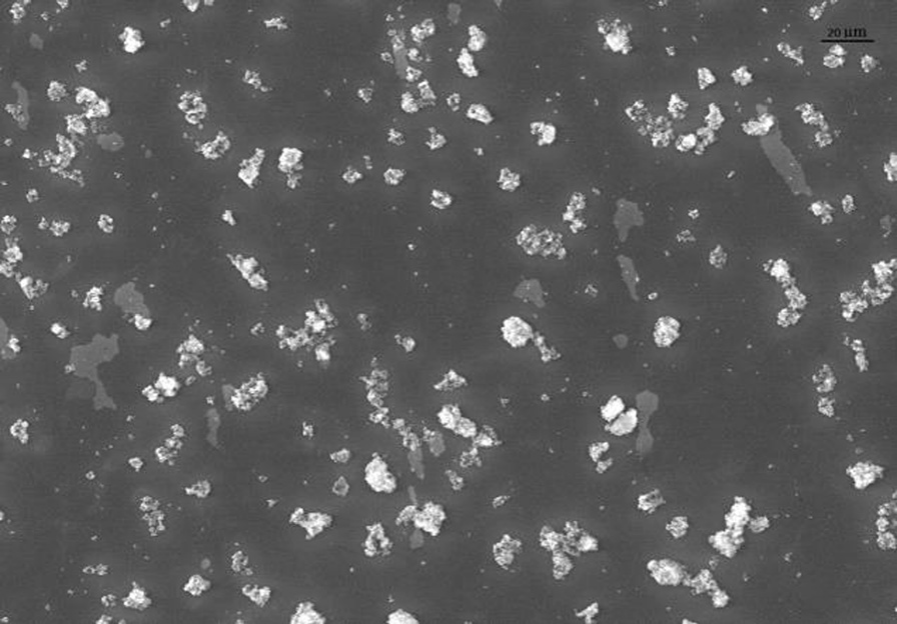
**TEM image of SiO**_**2**_ **nanoparticles.**

Figure 1 shows the average diameter of nano-SiO_2_ in solution was larger than the advertised diameter 30 nm, 0.0516 mV Zeta-potential, 0.404 uniformity and good particle diameter distribution. The microscraph also indicated that the particle morphology of nano-SiO_2_ was spherical shape. The imaged NP suspensions were dry in the high vacuum conditions in the transmission electron microscopy, so the transmission electron microscopy micrographs cannot provide an accurate presentation of the aqueous NP dispersion in the culture medium. Therefore, dynamic light scattering was utilized for evaluate particle size distribution in aqueous suspensions [26].

### Effects of SiO_2_ NPs on plant height and biomass

**Figure 2:**
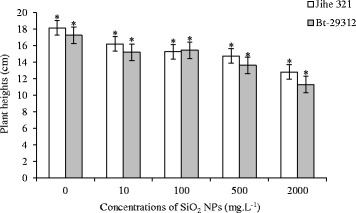
**Effects of SiO**_**2**_ **NPs concentrations on plant height.** The value was given as means ± SD. The (*) was represented significant difference (p <0.05) between control and SiO2 NPs treatments at the same cotton, and the (*) above the line segment was represented significant difference (p <0.05) between cultivars at the same SiO_2_ NPs concentration.

Figure 2 illustrates that the plant height was significantly different (p <0.05) between control and SiO_2_ NPs treatments in both Bt-transgenic and non-transgenic cotton. The plant heights of both non-transgenic and Bt-transgenic cotton were 18.14 and 17.25 cm as maximum at control treatment, respectively; and the height decreased with the increasing SiO_2_ NPs concentrations. The SiO_2_ NPs affected slightly on the plant height of non-transgenic and Bt-transgenic cotton when were treated by the below 500 mg.L^−1^ SiO_2_ NPs, but was obviously affected under 2000 mg.L^−1^ SiO_2_ NPs that the plant height of non-transgenic and Bt-transgenic cotton were minimum with 12.81 and 11.28 cm, respectively. These results are in contrast with the studies of Xuguang Li *et al.* [27]*,* which reported that the average plant height of Bt-transgenic and non-transgenic cotton were not affected by CeO_2_ nanoparticles. This suggested that the various nanomaterials have different effects on the same cultivars. On the other hand, the plant height of non-transgenic cotton was higher than Bt-transgenic cotton in both control treatment and almost SiO_2_ NPs treatments, but no significantly different were noticed. These results are in agreement with study of Mayee *et al.* [21] that non-transgenic cotton was developed faster than Bt-transgenic cotton.

**Figure 3:**
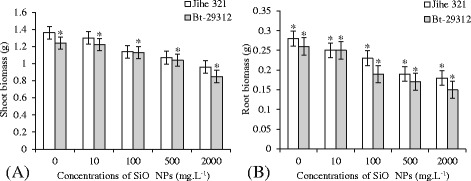
**Effects of SiO**_**2**_ **NPs concentrations on shoot and root biomasses.** The value was given as means ± SD. The (*) was represented significant difference (p <0.05) between control and SiO_2_ NPs treatments at the same cotton. Without the (*) above the line segment was represented no significant difference between cultivars at the same SiO_2_ NPs concentration.

The effects of SiO_2_ NPs concentrations on the root biomass were determined. It is clear from Figure 3B, which there was significantly different (p <0.05) between control and SiO_2_ NPs treatments at same cultivar root biomass. In addition, both of the root biomass of non-transgenic and Bt-transgenic cotton decreased along with the increased of SiO_2_ NPs concentrations. After 21 days treated with SiO_2_ NPs concentrations, the maximum of non-transgenic root biomass was 0.28 g at control treatment, 0.25, 0.23 and 0.19 g at 10, 100 and 500 mg.L^−1^ SiO_2_ NPs treatments, respectively; and 0.18 g as minimum under 2000 mg.L^−1^ SiO_2_ NPs treatment. Similarly, the Bt-transgenic root biomass were 0.26 and 0.15 g as maximum and minimum at control treatment and 2000 mg.L^−1^ SiO_2_ NPs treatment, respectively. These results are in agreement with previous study of Rao and Shekhawat [28]. The shoot biomass of non-transgenic and Bt-transgenic cotton decreased with the increase of SiO_2_ NPs concentrations, but the significance (p <0.05) was only noticed in Bt-transgenic shoot biomass (Figure 3A). The maximum shoot biomass of non-transgenic and Bt-transgenic cotton were 1.36 and 1.24 g at the control treatment, and 0.96 and 0.85 g as minimum when treated by 2000 mg.L^−1^ SiO_2_ NPs, respectively. On the other hand, no significant difference was noticed in the shoot biomass and root biomass between non-transgenic and Bt-transgenic cotton in both of control treatment and SiO_2_ NPs treatments. However, non-transgenic cotton shoot and root biomass were higher than Bt-transgenic cotton in both control treatment and SiO_2_ NPs treatments. This suggested that non-transgenic cotton was developed better than Bt-transgenic cotton, and Bt-transgenic cotton was more affected by SiO_2_ NPs than non-transgenic cotton. This result is in agreement with previous study of Xuguang Li *et al.* [27], who indicated that Bt-transgenic cotton is more sensitive to CeO_2_ nanoparticles than its parental non-transgenic cotton.

### Effects of SiO_2_ NPs concentration on nutrient contents in shoots and roots

**Figure 4:**
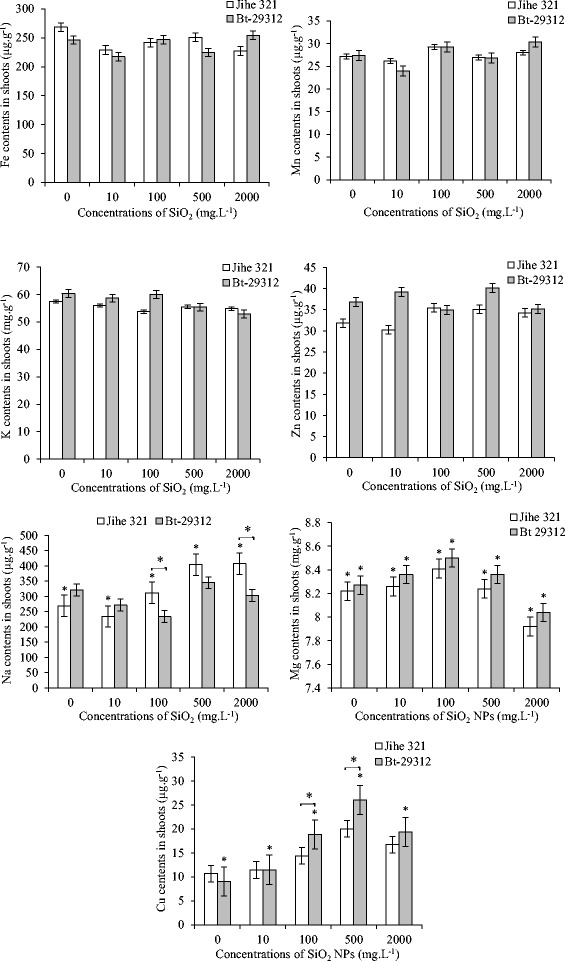
**Effect of SiO**_**2**_ **NPs concentrations on nutrient contents in shoots.** The value was given as means ± SD. The (*) was represented significant difference (p <0.05) between control and SiO_2_ NPs treatments at the same cotton, and the (*) above the line segment was represented significant difference (p <0.05) between cultivars at the same SiO_2_ NPs concentration.

**Figure 5:**
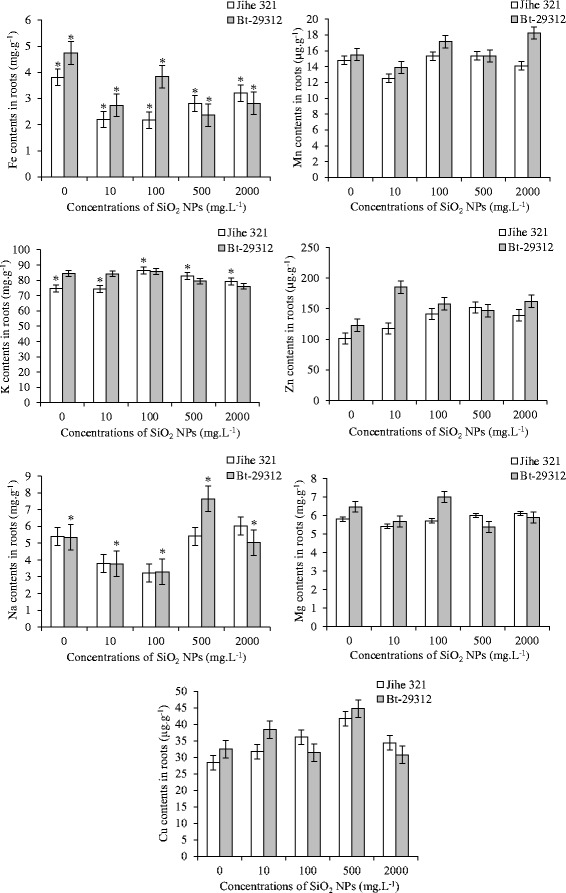
**Effect of SiO**_**2**_ **NPs concentrations on nutrient contents in roots.** The value was given as means ± SD. The (*) was represented significant difference (p <0.05) between control and SiO_2_ NPs treatments at the same cotton, and the (*) above the line segment was represented significant difference (p <0.05) between cultivars at the same SiO_2_ NPs concentration.

The nutrient elements in the shoots and roots of non-transgenic and Bt-transgenic cotton such as Fe, Mn, K, Zn, Na, Mg, and Cu were impacted by SiO_2_ NPs after 21 days, which was showed in Figure 4 and Figure 5.

Figure 4 showed that the contents of Fe, Mn, K and Zn in the shoots were not significantly differrent between non-transgenic and Bt-transgenic cultivars, and between control and SiO_2_ NPs treatments. Similar results were observed in Mn, Zn, Mg and Cu contents in roots; while Fe content was significantly different (p <0.05) in between cultivars, and between control and SiO_2_ NPs treatments. As shown in Figure 5, Fe content in Bt-transgenic roots were significantly higher at control treatment and treated with 10 and 100 mg.L^−1^ SiO_2_ NPs, but significantly lower than non-transgenic cotton at 500 and 2000 mg.L^−1^ SiO_2_ NPs. In comparison to the control, Mg content in shoots of non-transgenic and Bt-transgenic cotton that treated with different concentrations of SiO_2_ NPs showed a significant difference (p <0.05), the content of Mg increased with concentration up to 100 mg.L^−1^, but decreased with concentration up from 100 mg.L^−1^ to 2000 mg.L^−1^. There was a significant difference (p <0.05) between non-transgenic and Bt-transgenic cotton in Cu contents in shoots at 100 and 500 mg.L^−1^ SiO_2_ NPs treatments, and Na contents in shoot at 100 and 2000 mg.L^−1^ SiO_2_ NPs treatments. In addition, the Na contents in shoots and K contents in roots of non-transgenic cotton was significantly different (p <0.05) between control and SiO_2_ NPs treatments, while the significant s (p <0.05) were noticed in the shoot Cu contents and Na contents in Bt-transgenic cotton roots when compared to SiO_2_ NPs treatments with the control treatment. It is clear from Figure 4, the Cu contents in shoot of Bt-transgenic and non-transgenic cotton increased with concentrations up and maximum at 500 mg.L^−1^ SiO_2_ NPs with 26.07 μg.g^−1^ and 20.03 μg.g^−1^ then decreased to 19.36 and 16.73 μg.g^−1^ at 2000 mg.L^−1^ SiO_2_ NPs, respectively. Furthermore, Cu contents in roots of non-transgenic and Bt-transgenic cotton were 41.47 and 44.8 μg.g^−1^ as maximum at 500 mg.L^−1^ and reduced to 34.47 and 30.75 μg.g^−1^ at 2000 mg.L^−1^ SiO_2_ NPs, respectively. The Na content in Bt-transgenic roots was 7.63 μg.g^−1^ as a maximum and was significantly higher than non-transgenic at 500 mg.L^−1^ SiO_2_ NPs. These results suggested that Bt-transgenic roots absorbed and transported more Cu to shoots than non-transgenic roots. However, Na was more absorbed by Bt-transgenic roots but with fewer transported to the shoots than non-transgenic.

**Figure 6:**
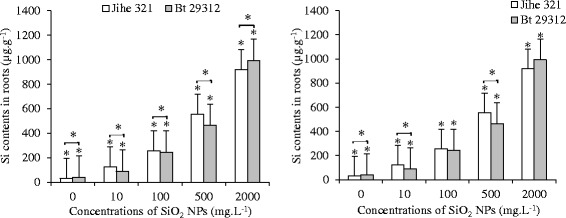
**Si contents in shoots and roots of non-transgenic and Bt-transgenic cottons.** The value was given as means ± SD. The (*) was represented significant difference (p <0.05) between control and SiO_2_ NPs treatments at the same cotton, and the (*) above the line segment was represented significant difference (p <0.05) between cultivars at the same SiO_2_ NPs concentration.

Figure 6 shows the Si contents in shoots and roots of non-transgenic and Bt-transgenic after 21 days exposure to SiO_2_ NPs. The Si contents in plant tissues increased with increasing SiO_2_ NPs concentrations. There were significant s (p <0.05) in root Si contents between control and SiO_2_ NPs treatments, and between Bt-transgenic and non-transgenic cotton at control treatment and 10, 500 mg.L^−1^ SiO_2_ NPs treatments. The Si content in Bt-transgenic cotton roots exposed to 2000 mg.L^−1^ SiO_2_ was 24.6 times higher than that of control, and 1.1 times higher than non-transgenic cotton. Similarly, Si contents in the shoots were significant s (p <0.05) between control and SiO_2_ NPs treatments, and between non-transgenic and Bt-transgenic cultivars. The Si contents in shoots of Bt-transgenic and non-transgenic cotton under treated with 2000 mg.L^−1^ SiO_2_ NPs was 14.9 times and 13.7 times higher than control treatments, respectively. These results illustrated that SiO_2_ NPs were absorbed by cotton plants and were transported to shoots from the root system. It is in agreement with previous studies of Wang *et al.* [29] that CuO NPs were uptaked by Maize through root system, and CeO_2_ NPs were transported from cotton root system to the shoots [27]. In addition, the uptake ability of SiO_2_ NPs differed between plant species that accumulated high, medium and low levels of Si were rice, cucumber and tomato, respectively [30]. According to the previous studies of Balakhnina *et al.* [31], Ma and Yamaji [32], Ma [33] reported that absorbed Si is beneficial for plant growth because its role in combating biotic and abiotic stress; and furthermore, Si was present in certain enzyme complexes promotes and protects photosynthesis [34]. However, Hoecke *et al.* [35] found that the toxicity of 12.5 and 27.0 nm Silica NPs (20.0 and 28.8 mg.L^−1^, respectively) to green alga was reduced growth by 20% after 72 h. Similarly, the green alga (*Scenedesmus obliquus*) was shown to reduce growth by 20% after 72 and 96 h under toxicity of 10 and 20 nm Silica particles (388.1 and 216.5 mg.L^−1^, respectively) [36]. In addition, mesoporous silica nanoparticles have been shown to penetrate tobacco mesophyll plant cells Torney *et al.* [37]. And the accumulation of FITC-labeled Si NPs in rice seedlings represents a future use for Si NPs in biolabeling of plant cells [38]. In one study, the Si NPs were uptaked into the root system of *Arabidopsis thaliana* [39]. According to Nair *et al.* [40] who indicated that plants are a potential pathway for bioaccumulation of NPs into the food chain and through ecosystems.

### Effects of SiO_2_ NPs on the nutrient concentrations in the xylem sap

Xylem sap plays an important role in the transport nutrient elements, minerals from the roots toward the leaves Marschner [41]. The effects of SiO_2_ NPs for the transport of nutrient elements in the xylem sap of Bt-transgenic cotton and non-transgenic cotton were assessed at the control and 2000 mg.L^−1^ SiO_2_ NPs treatments. The results showed that the concentrations of Cu and Zn in the xylem sap were significant s (p <0.05) between non-transgenic and Bt-transgenic cotton in both control and 2000 mg.L^−1^ SiO_2_ NPs treatments. Specially, the Cu and Zn concentrations of Bt-transgenic cotton were higher than non-transgenic cotton at the same treatment conditions. The Bt-transgenic cotton has 60.64 ng.mL^−1^ Cu and 207.55 ng.mL^−1^ Zn concentrations at the control treatment, while the non-transgenic cotton just only has 37.97 ng.mL^−1^ and 148.1 ng.mL^−1^, respectively. And under 2000 mg.L^−1^ SiO_2_ NPs, the Cu and Zn concentrations of Bt-transgenic cotton were 67.88 ng.mL^−1^ and 177.31 ng.mL^−1^ but only 34.33 ng.mL^−1^ Cu and 139.51 ng.mL^−1^ Zn in the non-transgenic cotton. The Cu and Zn concentrations in the xylem sap of non-transgenic and Bt-transgenic cotton were not significant between the control treatment and 2000 mg.L^−1^ SiO_2_ NPs treatment. This illustrated that the toxicity of SiO_2_ NPs had no effects on the concentrations of Cu and Zn in the xylem sap of non-transgenic and Bt-transgenic cotton.

**Figure 7:**
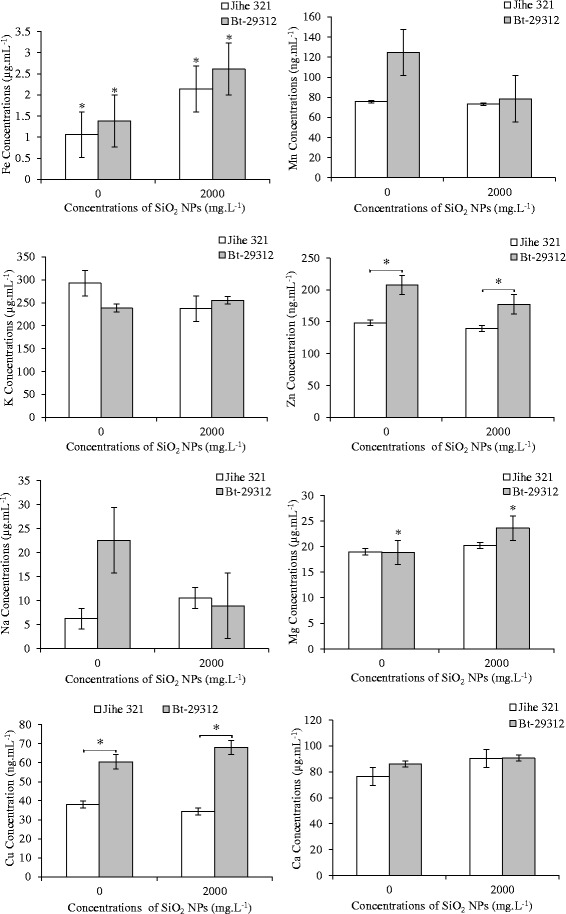
**Effects of SiO**_**2**_ **NPs concentrations on the nutrient concentrations in xylem sap of non-transgenic and Bt-transgenic cottons.** The value was given as means ± SD. The (*) was represented significant difference (p <0.05) between control and SiO2 NPs treatments at the same cotton, and the (*) above the line segment was represented significant difference (p <0.05) between cultivars at the same SiO2 NPs concentration.

Figure 7 indicates that the Mn, K, Na and Ca concentrations in the xylem sap were not significant s between the Bt-transgenic and non-transgenic cotton, and between the control treatment and 2000 mg.L^−1^ SiO_2_ NPs treatment. It indicates that SiO_2_ NPs had no effects on the concentrations of Mn, K, Na and Ca in the xylem sap of non-transgenic and Bt-transgenic cotton. In contrast to the above nutrient elements, the Mg and Fe concentrations in the xylem sap of non-transgenic and Bt-transgenic cotton were obviously impacted by the SiO_2_ NPs. Significant s (p <0.05) were noticed at Fe concentration in the xylem sap of non-transgenic and Bt-transgenic cotton between the control treatment and 2000 mg.L^−1^ SiO_2_ NPs treatment. As shown in Figure 7 that the Fe concentrations of Bt-transgenic cotton were significantly higher than non-transgenic cotton at same treatment conditions; and 2.14 μg.mL^−1^ and 2.61 μg.mL^−1^ Fe in the xylem sap of non-transgenic and Bt-transgenic cotton at 2000 mg.L^−1^ SiO_2_ NPs treatment were significantly higher than 1.06 μg.mL^−1^ and 1.38 μg.mL^−1^ at the control treatment, respectively. On the other hand, the Mg concentrations were significant s (p <0.05) between control and 2000 mg.L^−1^ SiO_2_ NPs treatments just only on Bt-transgenic xylem sap. Under 2000 mg.L^−1^ SiO_2_ NPs condition that the concentration of Mg was 23.59 μg.mL^−1^, whereas only 18.84 μg.mL^−1^ at the control treatment. These data showed that the SiO_2_ NPs promoted the transport of Mg in Bt-transgenic xylem sap and Fe in both the xylem sap of non-transgenic and Bt-transgenic cotton. However, no significant s was noticed in the concentrations of Mg and Fe in the xylem sap between Bt-transgenic and non-transgenic cotton. These results are in agreement with previously study of María José Gonzalo *et al.* [42], who indicates that Si was positively affected on Fe uptake and transport in soybean (*Glycine max*) and cucumber (*Cucumis sativus*). And according to Marmiroli *et al.* [9], Silicon was played as an important role in uptake and translocation of Arsenic in tomato (*Solanum lycopersicum L.*).

**Figure 8:**
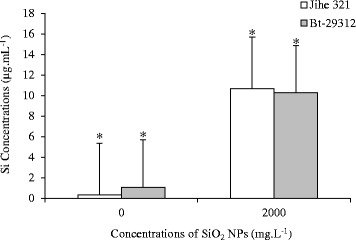
**Si concentrations in xylem sap of non-transgenic and Bt-transgenic cottons.** The value was given as means ± SD. The (*) was represented significant difference (p <0.05) between control and SiO2 NPs treatments at the same cotton, and the (*) above the line segment was represented significant difference (p <0.05) between cultivars at the same SiO2 NPs concentration.

**Figure 9:**
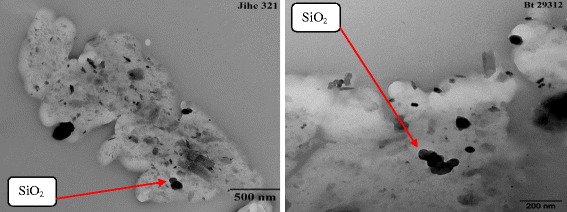
**TEM images of non-transgenic and Bt-transgenic cotton xylem sap.**

As shown in Figure 8, the Si concentrations in the xylem sap of non-transgenic and Bt-transgenic cotton were significant s (p <0.05) between the control and SiO_2_ NPs treatments at same cultivars. Under 2000 mg.L^−1^ SiO_2_ NPs treatment, Si levels in the xylem sap of non-transgenic and Bt-transgenic cotton were 10.68 μg.mL^−1^ and 10.28 μg.mL^−1^, respectively. Otherwise, SiO_2_ NPs were present in the xylem sap (Figure 9) as examined by transmission electron microscopy (TEM) showing that the SiO_2_ NPs were transported from roots to shoots via xylem sap. Zhang *et al.* [43] reported that Ceria NPs could be transferred from the roots to shoots in cucumber plants, and C_70_ NPs were entered into the roots and then be transported to the stem and leaves of rice plants [44]. These results suggested that SiO_2_ NPs were uptaked by cotton plants, which is consistent with previous reports of Xuguang Li *et al.* [27]; Shi *et al.* [45]; Lopez-Moreno *et al.* [46,47]. However, no significant difference were noticed between the Si contents in the xylem sap of the non-transgenic and Bt-transgenic cotton at both control treatment and 2000 mg.L^−1^ SiO_2_ NPs treatment. The TEM images showed that SiO_2_ NPs were in both the xylem sap of non-transgenic and Bt-transgenic cotton, this illustrated that SiO_2_ NPs were transported from roots to shoot via xylem sap. This study is in agreement with the previous study of Wang *et al.* [48], who reported that xylem sap could be a carrier for CuO NPs transportion in the maize (*Zea mays L.*).

### Effects of SiO_2_ NPs on the enzyme activities in the roots

**Figure 10:**
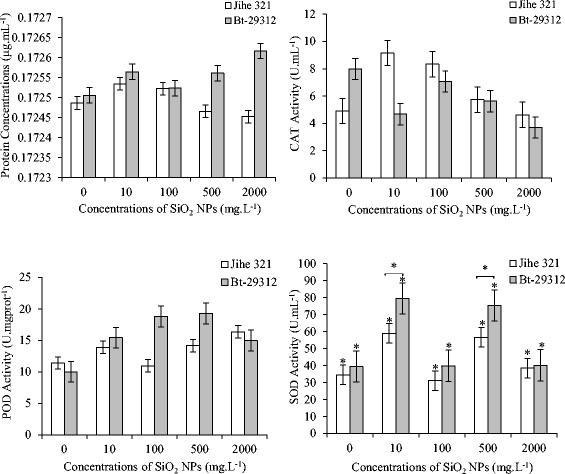
**Effects of SiO**_**2**_ **NPs concentrations on the enzymes activities of non-transgenic and Bt-transgenic cottons.** The value was given as means ± SD. The (*) was represented significant difference (p <0.05) between control and SiO2 NPs treatments at the same cotton, and the (*) above the line segment was represented significant difference (p <0.05) between cultivars at the same SiO2 NPs concentration.

It can be seen from Figure 10 that no significant s were observed in protein contents, CAT and POD activities between non-transgenic and Bt-transgenic cotton cultivars, and between the control and SiO_2_ NPs treatments. The protein contents in non-transgenic cotton roots decreased with the increasing SiO_2_ NPs concentrations from 10 to 2000 mg.L^−1^, whereas it was increased along SiO_2_ NPs concentrations up to 2000 mg.L^−1^ in Bt-transgenic cotton roots. CAT activities in the roots of non-transgenic and Bt-transgenic cotton were obviously decreased with the increasing of SiO_2_ NPs concentrations. Bt-transgenic cotton roots had 7,99 U.mL^−1^ and 3.70 U.mL^−1^ were maximum and minimum CAT activities at control and 2000 mg.L^−1^ SiO_2_ NPs treatments, respectively. Similarly, the activities of non-transgenic roots CAT were 9.15 U.mL^−1^ under 10 mg.L^−1^ SiO_2_ NPs treatment and decreased to 4.63 U.mL^−1^ at 2000 mg.L^−1^ SiO_2_ NPs treatment. These results are in agreement with previous study of Ali Riahi-Madvar [49] who investigated the effect of nanoparticles on the *Triticum aestivum*. According to Mukherjee *et al.* [50], the CAT acitivity in green peas (*Pisum sativum L*) was decreased by ZnO NPs concentrations, and Zhao *et al.* [51] reported a decrease in CAT activity in corn plants grown in organic soil treated with 400 mg.kg^−1^ of ZnO NPs. Thus, it is indicated that SiO_2_ NPs was negatively affected on the activities of CAT in the roots of non-transgenic and Bt-transgenic cotton. However, when treated by SiO_2_ NPs concentrations, activities of POD enzyme in the roots of non-transgenic and Bt-transgenic cotton were higher in comparision to the control treatment. And POD activities increased with SiO_2_ NPs concentration up to 500 mg.L^−1^ and decreased at 2000 mg.L^−1^. This suggested POD activities were stimulated by SiO_2_ NPs with concentrations lower than 500 mg.L^−1^ at both non-transgenic and Bt-transgenic cotton. As show in Figure 10 that the root SOD activities of non-transgenic and Bt-transgenic cotton were significantly different (p <0.05) between control and SiO_2_ NPs treatments; and the significant s (p <0.05) were also observed in SOD activities between non-transgenic and Bt-transgenic cotton at 10 and 500 mg.L^−1^ SiO_2_ NPs treatments. The maximum SOD activities of non-transgenic and Bt-transgenic cotton were 58.98 U.mL^−1^ and 79.51 U.mL^−1^ at 10 mg.L^−1^ SiO_2_ NPs treatments, respectively; but the lower results were similar under 100 and 2000 mg.L^−1^ SiO_2_ NPs treatments and control treatment. These results illustrated the activity of antioxidant enzymes were significantly decreased at the highest SiO_2_ NPs treatment concentration (Ali Riahi-Madvar, [49]).

### Effects of SiO_2_ NPs on the hormone concentrations in the roots

**Figure 11:**
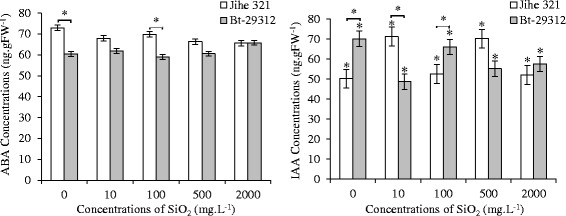
**Effects of SiO**_**2**_ **NPs concentrations on ABA and IAA hormone concentrations in the roots of non-transgenic and Bt-transgenic cottons.** The value were given as means ± SD. The (*) was represented significant difference (p <0.05) between control and SiO2 NPs treatments at the same cotton, and the (*) above the line segment was represented significant difference (p <0.05) between cultivars at the same SiO2 NPs concentration.

Indole-3-acetic acid (IAA) and abscisic acid (ABA) are plant hormones with contrasting biological functions. Whereas IAA stimulates growing processes such as cell elongation and division, ABA controls plant senescence and responses to stress [52,53]. The effects of SiO_2_ NPs on the ABA and IAA hormones of non-transgenic and Bt-transgenic cotton were showed in Figure 11.

ABA is mainly produced from the roots, and part of the ABA in roots is transported to leaves (Hezhong [54]). The ABA content in the roots was significantly different (p <0.05) between non-transgenic and Bt-transgenic cotton at control and 100 mg.L^−1^ SiO_2_ NPs treatments. The concentration of ABA in non-transgenic roots was obviously higher than Bt-transgenic cotton at control treatment and treated with 10, 100, 500 mg.L^−1^ SiO_2_ NPs; and it was 72.98 ng.gFW^−1^ as maximum at the control treatment and minimum under 2000 mg.L^−1^ SiO_2_ NPs (65.57 ng.gFW^−1^), whereas the ABA content in the roots of Bt-transgenic cotton was 65.80 and 59.01 ng.gFW^−1^ as maximum and minimum ABA levels at 2000 and 100 mg.L^−1^ SiO_2_ NPs treatments, respectively. Comparing the control treatment with SiO_2_ NPs treatments we found that the ABA concentration in the roots was not significant s in the same cultivars. Similarly, the significant s (p <0.05) were noticed in IAA concentration between non-transgenic and Bt-transgenic cotton at control treatment, 10, and 100 mg.L^−1^ SiO_2_ NPs treatments. The IAA contents of non-transgenic roots were obviously higher than Bt-transgenic under 10 and 500 mg.L^−1^ SiO_2_ NPs treatments, but lower at the control treatment, 100 and 2000 mg.L^−1^ SiO_2_ NPs treatments. From the Figure 11(b) it is clear that the concentrations of IAA in non-transgenic and Bt-transgenic roots were significant s (p <0.05) between the control treatment and SiO_2_ NPs treatments.

### Distribution of SiO_2_ in the cotton roots

**Figure 12:**
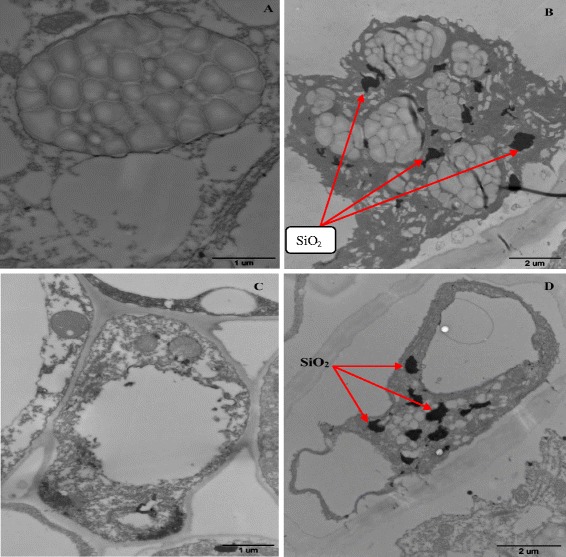
**TEM images of root sections of non-transgenic cotton (A-B) and Bt-transgenic cotton (C-D) after 21 days treated with SiO2 NPs. A** and **C** were TEM images of nontransgenic and Bt-transgenic cotton roots without SiO2 NPs treatments. **B** and **D** were TEM images of non-transgenic and Bt-transgenic cotton roots treated with 2000 mg.L^−1^ SiO_2_ NPs.

Figure 12 A-C shown in TEM images that root sections of Bt-transgenic cotton and non-transgenic cotton in the control treatment were free particle adherence. However, the absorption of SiO_2_ nanoparticles and their aggregation in the roots of non-transgenic and Bt-transgenic cotton were evident (Figure 12B-D). TEM images of the cross root sections of Bt-transgenic cotton and non-transgenic cotton show the presence of dark dots (particles) in the endodermis and vascular cylinder under 2000 mg.L^−1^ SiO_2_ NPs treatment. One or several nanoparticles could be identified in the dark dots covered by cytoplast as showed by higher magnification TEM image [55-57]. Most of SiO_2_ NPs were found on the root outer epidermis and only a few were located in intercellular spaces. These results illustrated that most of SiO_2_ NPs were located on root surface and only a very small amount of NPs could penetrate roots [27,43]. In addition, content of Si accumulated in the root of Bt-transgenic cotton and non-transgenic cotton were showed in Figure 6 and the Si content in the Bt-transgenic roots were higher than non-transgenic when treated with 2000 mg.L^−1^ SiO_2_ NPs, it suggests that SiO_2_ NPs was more easily penetrated into the root of Bt-transgenic cotton than non-transgenic cotton. Thus, it can be concluded that the SiO_2_ nanoparticles could enter into non-transgenic and Bt-transgenic roots, and be more potential harmful to Bt-transgenic cotton than non-transgenic cotton.

## Conclusions

In summary, SiO_2_ nanoparticles had significant influence on Bt-transgenic shoot biomass, cotton height and root biomass of both non-transgenic and Bt-transgenic cotton. The contents of nutrient elements such as Cu, Mg in shoots, Na in roots of Bt-transgenic cotton were significantly affected by SiO_2_ NPs; and more than non-transgenic cotton. We found that SiO_2_ NPs promoted the transport of Mg in Bt-transgenic xylem sap and Fe in both the xylem sap of non-transgenic and Bt-transgenic. In addition, the SOD activity and IAA concentration of both non-transgenic and Bt-transgenic cotton were significantly affected by SiO_2_ NPs. The Si contents increased with concentration up to 2000 mg.L^−1^ SiO_2_ NPs in both shoots and roots of Bt-transgenic cotton. And the Si level in the roots of Bt-transgenic cotton exposed to 2000 mg.L^−1^ SiO_2_ was 24.6 times higher than that of control treatment, and 1.1 times higher than non-transgenic cotton. Furthermore, SiO_2_ NPs were presented in the xylem sap (Figure 9) as examined by transmission electron microscopy (TEM) showing that the SiO_2_ NPs were transported from roots to shoots via xylem sap in both non-transgenic and Bt-transgenic. To our knowledge, this is the first report of the transportation of SiO_2_ NPs via xylem sap within Bt-transgenic cotton and non-transgenic cotton. This study provides direct evidence for the bioaccumulation of SiO_2_ NPs in plants, which shows the potential risks of SiO_2_ NPs impact on food crops and human health.

## Methods

### Characterisation of SiO_2_ nanoparticles

SiO_2_ nanoparticles (NPs) with average diameter of 30 ± 10 nm, were purchased from Sigma-Aldrich Chemical Company (St. Louis, MO, USA). Other chemicals were analytical grade and purchased from Beijing Chemical Plant. The aqueous suspensions of the studied nano particles have been previously characterized by the scaning electron microscopy [58]. SiO_2_ NPs stock dispersion (4000 mg.L^−1^) was prepared in filtered (0.45 μm) nanopure water (Millipore, resistivity >18.2 MΩ.cm^−1^). SiO_2_ NPs were dispersed in deionized water and sonicated (DEX® 130, 130 Watts, 20 kHz, Newtown, CT) at 70% amplitude during 15 mins before dropped on a Cu grid for TEM observation. The TEM images were obtained with JEM 200CX, Japan. The size distribution of SiO_2_ NPs was analyzed with dynamic light scattering (DLS) by a Coulter NicompTM 380 ZLS Paticle size analyzer (Santa Barbara, CA, USA) after dispersed in deionized water and sonicated for 30 mins.

### Cultivation of cotton and exposure

Transgenic cotton (Bt-29312) and non-transgenic cotton (Jihe 321) were given from Chinese Academy of Agricutural Sciences. Experimental cotton seeds were randomly selected and sterilized in (30%) H_2_O_2_ for 15 minutes, rinsed with deionized water, and then immersed in deionized water for 12-15 h before germination in sterilized, moist sand. After 7 days, similar seedlings of Bt-29312 and Jihe 321 were selected and transplanted to the 4.0 L pots containing 3.0 L nutrient solution, with two plants per pot. The nutrient solution was made following previously studies of Xuguang Li *et al*. [27] and 4 days/times periodly changed, after transplant 14 days SiO_2_ NPs were added into nutrient solution pots with different concentrations of 0, 10, 100, 500, and 2000 mg.L^−1^ respectively, each concentrations were repeated 4 times. SiO_2_ NPs were dispersed in deionized water and was ultrasonicated for 30 minutes using the ultrasonic clearner (KQ3200DE).

Experiments were repeated 3 times and were carried out from the March to April, 2014 in the greenhouse at China Agricultural University, and the aeration was maintained 24 h/24 h throughout the experiments.

### Measurement of biomass and plant height gain

The height of cotton was determined with the centimeter measurement from the growing point to cotyledon node. The plant height gain was determined following the formula: *ΔH* = *H*_*n*_ − *H*_*o*_ (cm).

Where: *H*_*o*_ was initial plant height that measured just before adding SiO_2_ NPs (cm).

*H*_*n*_ was the plant height that measured after 21 days treated with SiO_2_ NPs (cm)

Biomass measurement: After treated with SiO_2_ NPs 30 days, cotton plants were thoroughly washed with flowing tap water, and then with deionized water. Roots and shoots were seperatedly dried at 80°C for 24-36 h until constant weight achieved in a fan-forced oven, and then were immediately weighted.

### Si and nutrient contents determination

The dried roots and shoots were seperatedly grinded to fine powder by the high-speed pulverizer, 20–30 mg/samples were soaked in 5 mL (98%) HNO_3_ for 24 h, then 3 mL H_2_O_2_ was added and digested by using an electricity plate at 180°C for 4-5 h until 1 mL of solution remained. After that, it was diluted with deionized water, the mineral elements concentrations and Si concentrations were determined with using ICP-MS (*Inductively Coupled Plasma Mass Spectrometry*) (DRC-II) and ICP-AES (*Inductively Coupled Plasma – Atomic emission spectroscopy*) (iCap 6000).

### Xylem sap collection and determination of Si and nutrient contents

Xylem sap was extracted following the methods previously described of Wang *et al.* [29]. At the cotton 8–9 leaves stage, xylem sap was collected from the roots and shoots just below the cotyledon. Each de-topped root system was placed in pressure chamber, which contain its nutrient solution and the cut surface was wiped with deionized water to remove disrupted cells and residual cell elements, then a flexible silicon tube was placed 5–10 mm over the stump, tied tightly and linked to a centrifugal tube. The pressure of champer was applied at 0.2-0.3 MPa for 15 minutes for collecting the xylem sap. Nutrient elements and Si contents were determined with using the ICP-MS (DRC-II) and ICP-AES (iCap 6000) methods.

### Measurement of enzyme activities

Antioxidant enzymes such as catalase (CAT), superoxide dismutase (SOD) and peroxidase (POD) were extracted following the methods previously described of Dong and Chin [59]. The CAT activity were determined by monitoring the degradation of H_2_O_2_ (extinction coefficient 39.4 mM cm^−1^) at 240 nm [60], and the activity of SOD were assayed using the inhibition of nitroblue tetrazolium reduction at 560 nm [61]. According to Zhang [62] peroxidase activity (POD) was determined by mornitoring the formation of guaiacol dehydrogenation product (extinction coefficient 6.39 mM cm^−1^) at 436 nm. The proteins content were measured by Bradford [63] method using bovine serum albumin (BSA) as the standard.

### Determination of plant hormone concentration

Purification and extraction of absisic acid (ABA) and indole-3-acetic acid (IAA) were determined by ELISA methods as described in Gawronska *et al*. [64], which provided by Professor BM Wang from China Agricultural University, Beijing, China.

### Transmission Electron Microscopy (TEM) observation

Fresh roots of Jihe 321 and Bt-29312 in the control treatments and treated with 2000 mg.L^−1^ SiO_2_ NPs after 21 days treatment that were thoroughly washed with deionized water. The root tissues were further observed by TEM to see SiO_2_ NPs entered the plant cells. Samples were prepared following standard procedures [65,66]. Cotton root samples were prefixed in 2.5% glutaraldehyde, washed in 0.1 mol.L^−1^ pH 7.0 phosphate buffer, post-fixed in 1% osmium tetrooxide, dehydrated in acetone, and infiltrated and embedded in epoxy resin. The root sections were cut for TEM using a microtome with a diamond knife.

### Data analysis

The data across treatment groups were analyzed using one-way analysis of variance (ANOVA) and Turkey’s HSD test, which was performed using the statistical package SPSS Version 20.0. The data were expressed as means ± standard deviation (SD), a confidence interval of 95% (p <0.05) was consider significant in all cases.

## Abbreviations

ABA: Absisic acid
ANOVA: Analysis of variance
BSA: Bovine serum albumin
CAT: Catalase
ELISA: Enzyme linked immuno sorbent assay
IAA: Indole-3-acetic acid
ICP-AES: Inductively coupled plasma – atomic emission spectroscopy
ICP-MS: Inductively coupled plasma mass spectrometry
NPs: Nanoparticles
POD: Peroxidase
SD: Standard deviation
SOD: Superoxide dismutase
TEM: Transmission electron microscopy
